# Reference‐Superimposed Reconstruction (RS‐Recon) for Arterial Spin Labeling

**DOI:** 10.1002/mrm.70540

**Published:** 2026-07-26

**Authors:** Jia Guo

**Affiliations:** ^1^ Department of Bioengineering University of California Riverside Riverside California USA

**Keywords:** arterial spin labeling, background suppression, image reconstruction, low‐SNR signal recovery, phase estimation

## Abstract

**Purpose:**

Conventional arterial spin labeling (ASL) reconstruction suffers from sign‐flipping errors and/or noise bias under strong background suppression (BS), limiting SNR optimization. We propose reference‐superimposed reconstruction (RS‐recon) to enable ideal BS and robust signal recovery.

**Methods:**

RS‐recon superimposes high‐SNR *M*_0_ *k*‐space data onto ASL *k*‐space before reconstruction, with subsequent reference subtraction. Four volunteers underwent 3T scanning with pulsed ASL, pseudo‐continuous ASL, and dual‐module velocity‐selective ASL across varying BS levels. RS‐recon integrated reconstruction pipelines were compared to standard magnitude (Mag), complex subtraction (ComplexSub), and complex (Complex) reconstruction pipelines. The performance was evaluated by metrics including ASL signal, temporal SNR (tSNR), artifacts, misalignment robustness, and generalized auto‐calibrating partially parallel acquisitions (GRAPPA) compatibility.

**Results:**

RS‐recon eliminated subtraction artifacts due to signal sign‐flipping, recovered negative ASL signals, and enabled ideal BS for maximizing SNR. It significantly improved ASL signal and tSNR in gray/white matter across all labeling methods and different BS conditions. RS‐recon corrected phase errors under low‐SNR conditions, preserved signal signs, maintained Gaussian noise distribution, remained robust against misalignments between the reference and the ASL images, and integrated well with GRAPPA. Mag + RS performance matched complex‐based (ComplexSub and Complex) RS pipelines and is recommended for its simplicity, compatibility, and robustness overall.

**Conclusion:**

RS‐recon offers a simple, robust strategy for ASL image reconstruction, resolving subtraction errors even with magnitude output and enabling optimal background suppression. It enhances signal fidelity, SNR, and phase accuracy while integrating seamlessly with existing reconstruction pipelines and parallel imaging.

## 1 Introduction

Conventionally, arterial spin labeling (ASL) [1] images, like most of MR images, are reconstructed with only the magnitude retained for its robustness, simplicity, and convenience to visualize. This works well under the assumption that the combined MR signals from tissue and arterial blood across the entire imaging volume are of the same sign under both the label and control conditions, for example, all positive, so that there is no sign‐flipping between the label and control conditions when magnitude images are, and ASL signals can be successfully extracted by subtraction without error. Due to the low intrinsic low SNR of ASL, background suppression (BS) [2] is strongly recommended whenever possible to improve the SNR [3] by reducing the tissue signal and associated signal fluctuations. When a strong BS is applied, the above assumption may no longer hold true, and ASL subtraction errors can occur.

To avoid such problems in magnitude reconstruction (Mag, Figure 1A), different approaches are adopted in practice, including: (1) using less aggressive BS, for example, keeping all tissue signals positive and on the order of ∼5%, so that the assumption above remains effective, as typical ASL signal amplitude is on the order of 1% of relaxed tissue signal; (2) performing complex subtraction (ComplexSub, Figure 1B) between the label and control *k*‐space data before image reconstruction to avoid sign‐flipping, and then often followed by temporal averaging and sum‐of‐square (SOS) coil combination to obtain magnitude ASL images; (3) performing a complex reconstruction (Complex, Figure 1C), and then project the raw ASL signals using the complex *M*_0_ images from the calibration scan as the reference, where the in‐phase signals yield ASL signals after subtraction and the out‐of‐phase signals are discarded as noise [4]. However, these methods are not ideal and have limitations. Approach 1 does not take the full advantage of BS and the BS optimization process is more constrained, both can result in suboptimal SNR. In ComplexSub, individual raw label and control images are discarded, making it difficult to perform motion correction if needed; the ASL signal sign information is lost, which may be informative; in addition, the noise is rectified in the magnitude output and the distribution is changed from Gaussian to Rician, introducing biases, especially if the temporal averaging is done after image reconstruction. In Complex, the ASL signals are likely more accurate with most of the noise removed in the projection process; however, when the MR signal is extremely weak, such as with a very strong BS, the complex reconstruction itself may be inaccurate due to low‐SNR phase estimation, and can lead to ASL signal detection errors, as reported previously [4].

**FIGURE 1:**
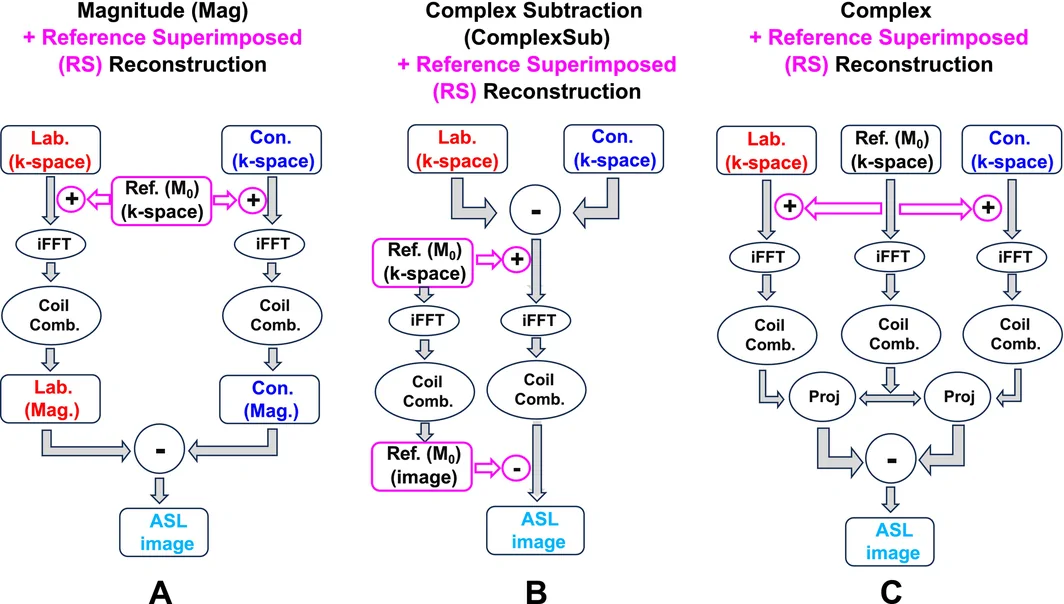
Diagrams of different reconstruction pipelines: (A) Magnitude reconstruction (Mag); (B) complex‐subtraction reconstruction (ComplexSub); (C) complex reconstruction (with signal projection); and the corresponding variants combined with reference‐superimposed (RS) reconstruction (highlighted in magenta).

Here we propose a novel reconstruction strategy for ASL that is simple and robust, and solves the above limitations of the existing reconstruction methods for ASL. It enables ideal BS (near‐zero tissue signals) to be used in practice for maximized SNR improvement from BS, without the concern of ASL subtraction errors due to sign‐flipping even with magnitude output. The strategy is described in details below, where we also provide a formalized and modular description of image reconstruction pipeline in ASL imaging in general.

## 2 Theory

This new strategy termed Reference‐Superimposed reconstruction (RS‐recon) takes advantage of the high‐SNR *M*_0_ calibration data that almost all ASL studies include as part of the acquisition, where the *k*‐space data of the *M*_0_ scan is superimposed to the *k*‐space data of the ASL acquisition through a linear combination: $S_{\mathrm{ASL},\mathrm{RS}-\text{recon}}=S_{\mathrm{ASL}}+\alpha \cdot S_{M0}$, where $S_{\mathrm{ASL}}$ and $S_{M0}$ are the original *k*‐space signals from the ASL and *M*_0_ scans, and α is a scaling factor defined by the user (see below). Then routine image reconstruction methods can be performed.

This simple strategy works in the following aspects:

1. Prevent signal sign‐flipping: adding large constant in‐phase signals from the reference scan ensures that the combined signals are of the same sign, preventing potential signal sign‐flipping during reconstruction.
2. Improve the SNR of phase estimation: the superimposed reference signal improves the accuracy of phase estimation, especially when the SNR is low, for example, under a strong BS where the MR signals are near zero. When phased‐array multi‐channel coil is used in image acquisition, accurate estimation of the relative phase from different coil channels is necessary for correct signal combination.
3. Provide high‐SNR coil sensitivity information: superimposing the reference signal embeds high‐SNR coil sensitivity information into the measured signal, which provides a convenient and robust estimation of coil sensitivity for adaptive coil combination, especially when dedicated coil sensitivity information is not available from a separate calibration scan.

The choice of α depends on the raw ASL signal, or BS, level. Here we define a BS factor β=1/(BSlevel), for example, a BS level of 10% corresponds to β=10. To prevent signal sign‐flipping, it requires α>1/β. For a phase estimation with improved accuracy (lower mean squared error, MSE), that is, $\frac{{\mathrm{MSE}}_{\mathrm{RS}}}{{\mathrm{MSE}}_{\text{orig}}}<1$, it needs to satisfy $\left(2-\beta^{2}\right)\alpha <2\beta$. For $\beta \geq \sqrt{2}$, the inequality holds for all positive α; for 1 $<\beta \leq \sqrt{2}$, it holds when $\alpha <\frac{2\beta }{\left(2-\beta^{2}\right)}$. The minimum of $\frac{{\mathrm{MSE}}_{\mathrm{RS}}}{{\mathrm{MSE}}_{\text{orig}}}$ is reached when α=β/2; however, given a higher β, a much lower α is needed to approximate the minimum, that is, the highest accuracy, as shown in Figure 2. For example, α values of around 0.5 and 0.2 are sufficient to offer significant accuracy improvement on the phase estimation with β of 10 and 20, respectively; while preventing signal sign‐flipping. Detailed derivation can be found in Supporting Information.

**FIGURE 2:**
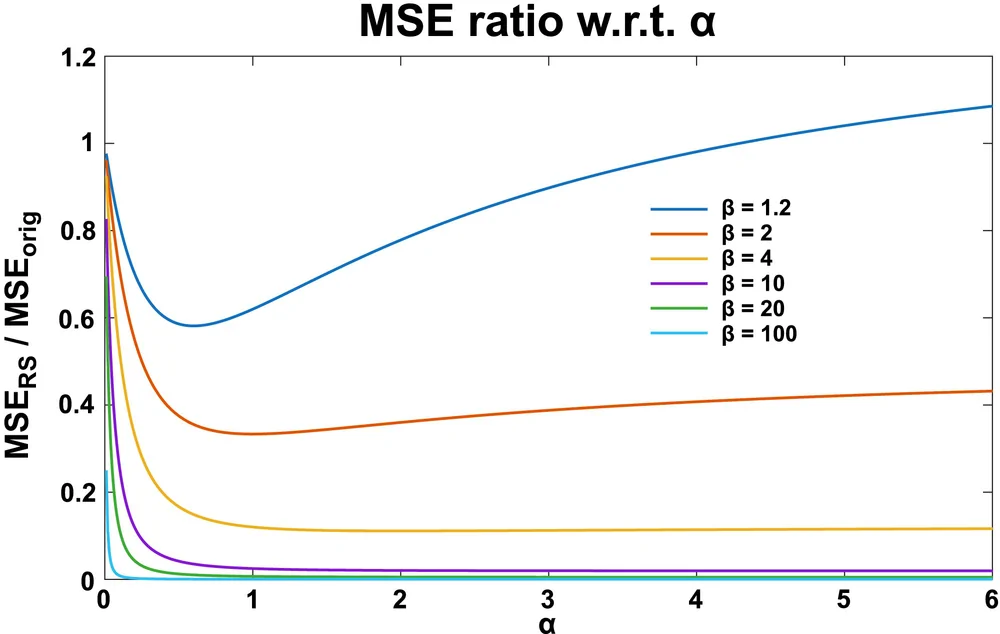
The ratio of MSE_RS_ to MSE_orig_ as a function of α, given different background suppression (BS) levels, that is different β values. For example, β=10 and β=100 correspond to 10% and nearly ideal BS, respectively.

In addition, because constant superimposed reference signals are added and then removed later, either through label/control subtraction or explicit subtraction of the reference image, RS‐recon does not change the temporal noise characteristics of the ASL acquisition, nor the CBF quantification. Note that adding a large constant value does not work, especially with multi‐channel coil combination, as the reference signal needs to be in‐phase with the signal under detection across the entire imaging volume.

To better describe how RS‐recon works with the existing ASL image reconstruction pipeline, we first describe the reconstruction pipeline in ASL in a modularized fashion by defining four major components:

1. Domain of subtraction: this is to differentiate whether the subtraction is performed in *k*‐space (conventionally referred to as complex subtraction) or image space, and not referring to the specific subtraction methods to yield perfusion‐weighted ASL signals, such as pair‐wise, surround [5], or sinc subtraction [6] methods.
2. Inverse Fourier transform (iFT): to convert the information from *k*‐space to image space.
3. Coil combination: such as SOS combination, adaptive combination, etc. The choice of coil combination method also affects what form the output can take. For example, only magnitude output form is available with SOS.
4. Output form: magnitude output and complex output with signal projection. Magnitude is the most widely used form of output. It is easy to visualize, but may cause subtraction error in ASL if signal sign‐flipping occurs. The complex output form allows differentiation of ASL signal of opposite signs and reduction of noise through signal projection, which we loosely described as the complex reconstruction method previously [4].

The ASL reconstruction pipeline can be considered as making different choices of these modules to form a reasonable combination. For example, a typical magnitude reconstruction (Mag) can use either SOS or adaptive coil combination and then take the magnitude output; a conventional ComplexSub reconstruction can be considered as a combination of complex subtraction in *k*‐space, iFT, SOS combination, and magnitude output; while an improved alternative pipeline can be a combination of complex subtraction in *k*‐space, iFT, adaptive coil combination, and complex output form, which allows complex signal projection for signed ASL signals.

Under this formalized description of ASL reconstruction pipeline, the concept of RS‐recon can be readily appreciated in the demonstration in Figure 1, where it can be implemented in combination with different existing reconstruction pipelines. Perfusion‐weighted images can be generated by label/control subtraction where the reference signal is canceled out when combined with Mag reconstruction; or by a similar projection and subtraction approach as in Complex; or by removing the added reference signal after image reconstruction when combined with ComplexSub. In addition, if subtraction is done in the image‐domain, the signed individual label/control images can be obtained by subtracting the reference image from the reconstructed raw ASL images. This allows image‐based motion correction at individual time points before the label/control subtraction.

## 3 Methods

### 3.1 Image Acquisition

To evaluate the RS‐recon strategy, four healthy volunteers (2F, 39.3 ± 5.9 years old) were scanned on a 3T scanner (MAGNETOM Prisma, Siemens Healthineers, Erlangen, Germany) with University of California Riverside IRB approval and participant consent. Scans covering all three types of ASL were performed at different levels of BS: (1) Pulsed ASL (PASL) using FAIR [7] and Q2TIPS [8], labeling duration (LD) = 0.7 s, post‐labeling delay (PLD) = 1.1 s; (2) Pseudo‐Continuous ASL (PCASL) [9], LD = 1.8 s, PLD = 1.8 s; (3) dual‐module velocity‐selective ASL (dm‐VSASL) [10] using sinc‐VSI pulse [11] with different labeling inflow times (see below). PASL and PCASL were included in a WIP sequence with two options (levels) of BS, referred to as the BS and BS‐strong conditions here, using 2 and 3 BS pulses in PASL, and 4 and 5 BS pulses in PCASL, respectively. The gray matter (GM) and white matter (WM) signals were ∼5% and ∼7% under the BS condition, and ∼3% and < 1% under the BS‐strong condition for PASL; and ∼10% and ∼10% under the BS condition, and ∼4% and ∼5% under the BS‐strong condition for PCASL, respectively. The in‐house developed dm‐VSASL sequence was scanned with three levels of BS using the inherent inversion of the two VSI labeling modules at different inversion times [4]: (1) TI_1_/TI_2_ = 1.6/0.4 s, tissue signals ∼4% in GM and ∼5% in WM; (2) TI_1_/TI_2_ = 1.65/0.35 s, tissue signals < 1% in GM and ∼2% in WM, and (3) TI_1_/TI_2_ = 1.65/0.35 s, tissue signals ∼−2% in GM and ∼−2% in WM. The *M*_0_ reference images were collected in the same ASL scan with the WIP sequence, and in a separate scan with the dm‐VSASL sequence. All data were acquired with the same set of readout parameters: a single‐shot 3D gradient‐ and spin‐echo (GRASE) echo‐planar imaging (EPI) readout with a matrix of 64 × 61 × 16 and a field of view of 220 × 220 × 64 mm without parallel‐imaging acceleration. The TR was 4 s for PASL, 5 s for PCASL, and 5.3 s for dm‐VSASL; the TE was 35 ms for all; and the number of label/control pairs was 21, 17, and 16 for PASL, PCASL and dm‐VSASL, respectively, corresponding to a similar total acquisition time. *T*_1_‐weighted anatomical images were also collected for region‐of‐interest (ROI) analysis.

To further understand the RS‐recon principle and evaluate its performance with respect to potential misalignment between the ASL and the reference scans, additional ASL scans were performed on two of the subjects with a 20‐channel head‐and‐neck receive coil (with 16‐channel head components in effect, referred to as the 16‐channel coil in the following text), using PCASL with the BS condition, and dm‐VSASL with TI_1_/TI_2_ = 1.6/0.4 s.

To test whether RS‐recon works with parallel imaging, ASL scans using a 32‐ch receive coil were performed on one of the subjects with a generalized auto‐calibrating partially parallel acquisitions (GRAPPA) [12] acceleration factor of 2 along the phase encoding direction, using PASL and PCASL with the BS‐strong condition, and VSASL with TI_1_/TI_2_ = 1.65/0.35 s.

### 3.2 Image Reconstruction and Analysis

The ASL image reconstruction strategies were examined in two different environments: (1) simpler and more generic in‐house implementations in MATLAB (R2025a, The MathWorks Inc., Natick, Massachusetts, USA), where different reconstruction pipelines can be compared; and (2) the retrospective reconstruction pipeline provided on the scanner, where the choice of reconstruction pipelines was limited but more sophisticated post‐processing was included, such as matrix resizing, apodization, proprietary adaptive coil combination with pre‐acquired coil sensitivity maps, etc., which generally resulted in better image quality.

For the in‐house reconstruction, the *k*‐space data were loaded in MATLAB using the mapVBVD toolbox [13], corrected for EPI timing errors, and then undergone reconstruction pipelines with different combination of the reconstruction components: (1) Mag, that is, image‐domain subtraction + SOS (or adaptive) coil combination + magnitude output; (2) complex, that is, image‐domain subtraction + adaptive coil combination + complex signal projection with signed real output; (3) ComplexSub, that is, *k*‐space subtraction + SOS (or adaptive) coil combination + magnitude output; and their corresponding counterparts with RS‐recon included. Note that only commonly used combinations of the reconstruction components were studied here for simplicity and demonstration purposes. The adaptive coil combination [14] was modified from a MATLAB implementation [15] to also allow the use of estimated sensitivity maps from the *M*_0_ images for improved reconstruction results.

For the on‐scanner retrospective reconstruction (software version XA30A) using RS‐recon, the *k*‐space data were loaded using twixtools [16] in a Python script, then the *k*‐space data of the reference *M*_0_ scan were added to the *k*‐space data of the ASL scans with α of 0.15 for dm‐VSASL data or 0.5 for WIP PASL and PCASL data. These values were chosen to benefit from the RS principles while avoiding data over‐ranging, as the on‐scanner reconstruction pipeline was scheduled to output the images with a multiplication factor of 5 with a limited dynamic range for the VSASL sequence, and a factor of 10 for the WIP sequence but with a wider dynamic range. Then the modified *k*‐space data were imported onto the scanner for retrospective reconstruction with adaptive coil combination, and complex (for dm‐VSASL only) or magnitude (for all ASL methods) output. The on‐scanner reconstruction time of the RS‐combined pipelines was the same as those without.

The *T*_1_‐weighted anatomical images were processed in the FSL toolbox [17] to generate GM and WM ROIs that were then co‐registered to the *M*_0_ images generated on the scanner for analysis in the local space. Necessary shifting and shrinking were applied on the ROIs for the in‐house reconstructed image analysis to account for the different in‐plane image matrix sizes: 64 × 61 (in‐house) versus 64 × 64 (on‐scanner).

ASL signals were obtained by pair‐wise subtraction, and the temporal SNR (tSNR) was calculated accordingly, except for some of the ComplexSub‐based pipelines where the tSNR was unavailable either because temporal averaging for improved SNR was done before coil combination and image output (pre‐averaging), or rectification of the MR signal and noise prevented unbiased tSNR calculation. Normalized tissue and ASL signals were calculated by dividing by the *M*_0_ image. As the Mag reconstruction pipeline is most widely used, the comparisons were focused on the Mag‐recon with SOS and adaptive coil combinations, and their RS‐recon counterparts for simplicity and visualization clarity. The ASL signal and tSNR were averaged in the GM and WM ROIs and compared, and the percentage improvement with RS‐recon were calculated across subjects, and tested for significance using paired *t*‐test with a significance threshold of 0.05, and Bonferroni correction when applicable. Analysis of variance (ANOVA) was performed on representative RS‐recon based pipelines of different types, that is Mag + RS (SOS), Mag + RS (adaptive coil combination with magnitude output), ComplexSub + RS (adaptive coil combination with projection), and Complex + RS (adaptive coil combination with projection).

To study RS‐recon's sensitivity to the misalignment between the reference and the ASL scans, a range of 3D diagonal displacements of 1, 2, 4, 6, 8, 12, 16, 24, and 32 mm were simulated on the *M*_0_ data, and then followed by RS‐recon in the in‐house reconstruction experiments. For simplicity, only Mag and Mag + RS were compared using the PCASL and dm‐VSASL data acquired with 16‐channel and 32‐channel coils. The results using Mag + RS without any displacement were used as the normalization reference to show the relative changes under different misalignment conditions.

For the on‐scanner image reconstruction with GRAPPA, Mag and Mag + RS were compared with adaptive coil combination and magnitude output.

## 4 Results

The reconstruction results on dm‐VSASL data using Mag and Complex and their counterparts with RS‐recon are shown in Figure 3. The dm‐VSASL results well demonstrated the performance differences as the tissue signals covered positive, near‐zero, and negative conditions. These images were reconstructed on‐scanner to take advantage of advanced reconstruction processing. As expected, the tissue signal sign information was lost with Mag reconstruction, while Mag + RS correctly recovered negative tissue signals even with SOS coil combination and magnitude output, yielding identical tissue images as the Complex and Complex + RS. The diminished and false “negative” ASL signals with Mag due to the signal sign‐flipping with strong BS (TI_1_/TI_2_ = 1.65/0.35 s) or negative tissue signals (TI_1_/TI_2_ = 1.7/0.3 s) were recovered with RS‐recon. Even when the tissue signals were all positive and relatively strong (∼5% with TI_1_/TI_2_ = 1.6/0.4 s), RS‐recon was able to recover an unexpected subtle ASL signal reduction, likely due to artifacts from fat. Comparing to Mag, most of the ASL signal detection errors due to sign‐flipping were resolved with Complex as expected. However, when the MR signal was extremely weak (TI_1_/TI_2_ = 1.65/0.35 s), that is, near zero, artifacts occurred, likely caused by inaccurate signal/phase detection during coil combination, as reported previously [4]. Such artifacts were clearly shown in the residual signal (noise) maps. When combined with RS‐recon, the artifacts were completely removed, yielding consistent ASL and residual signal maps regardless of the BS conditions or tissue signal signs, as shown in Figure 3E.

**FIGURE 3:**
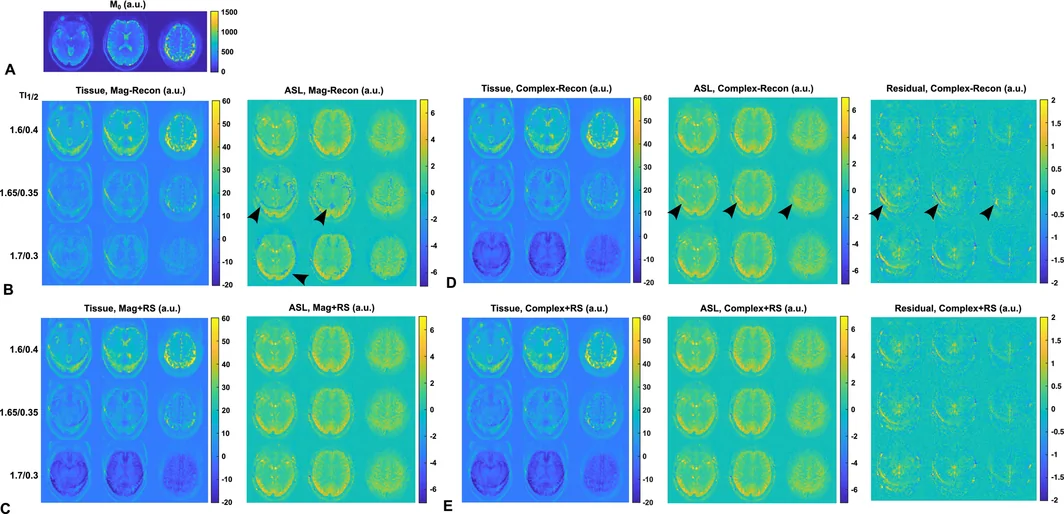
Examples of reconstructed dm‐VSASL images at different BS levels, using Mag‐recon and Complex‐recon, and their RS‐recon counterparts. (A) *M*_0_ reference images; (B) tissue and ASL images using Mag reconstruction; (C) tissue and ASL images using Mag + RS reconstruction; (D) tissue and ASL images using Complex reconstruction; (E) tissue and ASL images using Complex + RS reconstruction. Black arrowheads indicate ASL artifacts due to sign‐flipping and inaccurate phase detection during coil combination; RS‐based reconstruction eliminated such artifacts, and preserved the signal signs.

Examples of the reconstruction results comparing Mag versus Mag + RS, and SOS (in‐house) versus adaptive coil combination (on‐scanner) in all three labeling methods are shown in Figure 4. Both the normalized ASL signal and tSNR maps showed that RS‐recon successfully preserved the tissue signal signs and corrected ASL subtraction artifacts across all labeling methods, even in PASL and PCASL with the BS condition where ASL signal detection errors were unexpected with relatively strong and positive tissue signals. Similar ASL signal and tSNR maps were obtained using SOS and adaptive coil combinations, where the latter should yield more accurate results. In the PASL results with strong BS, it was clear that the sign‐flipping in WM due to suboptimal BS caused the artifacts (negative ASL signal); while RS‐recon fully recovered the ASL signal throughout the brain, demonstrating its robustness under different BS conditions.

**FIGURE 4:**
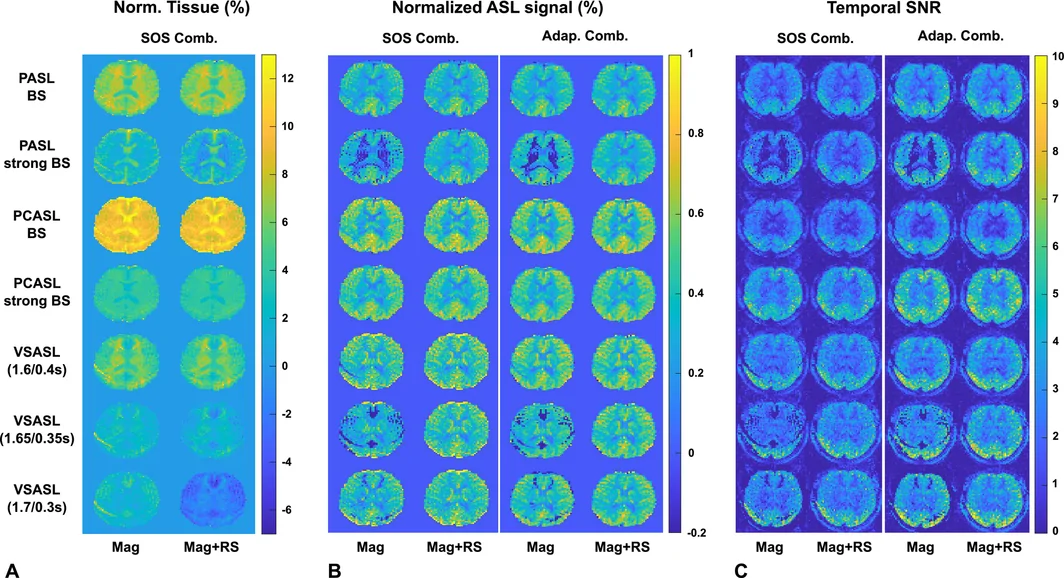
Examples of reconstructed PASL, PCASL, and dm‐VSASL images at different levels of background suppression, using Mag and Mag + RS reconstruction with SOS (in‐house) and adaptive coil combination (on‐scanner). (A) Normalized tissue maps showing the BS levels; (B) normalized ASL images; (C) temporal SNR maps. RS‐based reconstruction effectively recovered ASL signals under both reconstruction settings. The image quality and tSNR were higher using adaptive coil combination and other processing in the on‐scanner environment.

Following the results in Figure 4, normalized ASL signal and tSNR in GM and WM using these pipelines, averaged across subjects, are summarized in Table 1. Note that the higher absolute tSNR values with the on‐scanner reconstruction were due to the additional post‐processing as mentioned earlier. The averaged percentage changes with RS‐recon included are shown in Figure 5. RS‐recon significantly improved the ASL signal and tSNR in GM and WM when combined with Mag‐recon in most of the labeling and BS conditions, despite the small number of subjects. Largest improvements were observed when subtraction errors in large areas were corrected by RS‐recon, for example, in PASL with suboptimal BS (the BS‐strong condition) and dm‐VSASL with TI_1_/TI_2_ = 1.65/0.35 s (nearly ideal BS). In PCASL small but consistent improvements using RS‐recon were also observed even when the BS was designed to work well with Mag. A more complete summary of normalized ASL signal and tSNR using different reconstruction pipelines, including ComplexSub and Complex‐recon based methods, is summarized in Tables S1 and S2.

**TABLE 1:** Normalized ASL signal and temporal SNR (tSNR) in gray matter (GM) and white matter (WM) using Mag and Mag + RS with different coil combination methods and reconstruction environments, averaged across subjects (mean ± standard deviation, std).

		Normalized ASL (%, mean ± std)				tSNR (mean ± std)			
		In‐house recon		On‐scanner recon		In‐house recon		On‐scanner recon	
ASL methods (BS conditions)		Mag (SOS)	Mag + RS (SOS)	Mag (Adap.)	Mag + RS (Adap.)	Mag (SOS)	Mag + RS (SOS)	Mag (Adap.)	Mag + RS (Adap.)
PASL (BS)	GM	0.346 ± 0.068	0.353 ± 0.066	0.328 ± 0.065	0.334 ± 0.064	1.875 ± 1.051	1.912 ± 1.059	2.758 ± 0.971	2.810 ± 0.985
	WM	0.274 ± 0.048	0.277 ± 0.046	0.255 ± 0.045	0.257 ± 0.045	1.416 ± 0.786	1.426 ± 0.784	2.181 ± 0.676	2.203 ± 0.678
PASL (BS‐strong)	GM	0.273 ± 0.067	0.339 ± 0.064	0.288 ± 0.064	0.319 ± 0.064	1.530 ± 0.827	1.729 ± 0.882	2.363 ± 0.753	2.558 ± 0.773
	WM	0.069 ± 0.021	0.265 ± 0.051	0.058 ± 0.024	0.245 ± 0.049	0.454 ± 0.274	1.335 ± 0.713	0.544 ± 0.249	2.112 ± 0.549
PCASL (BS)	GM	0.451 ± 0.090	0.458 ± 0.090	0.453 ± 0.089	0.460 ± 0.090	1.737 ± 0.721	1.741 ± 0.724	2.352 ± 0.606	2.368 ± 0.612
	WM	0.359 ± 0.058	0.365 ± 0.059	0.355 ± 0.058	0.360 ± 0.059	1.577 ± 0.660	1.579 ± 0.665	2.273 ± 0.462	2.306 ± 0.467
PCASL (BS‐strong)	GM	0.403 ± 0.079	0.410 ± 0.076	0.415 ± 0.076	0.419 ± 0.076	2.369 ± 1.158	2.392 ± 1.149	3.647 ± 0.837	3.680 ± 0.830
	WM	0.327 ± 0.055	0.332 ± 0.054	0.330 ± 0.051	0.332 ± 0.051	1.886 ± 0.956	1.891 ± 0.954	2.999 ± 0.633	3.034 ± 0.620
Dm‐VSASL (1.6/0.4 s)	GM	0.393 ± 0.077	0.426 ± 0.078	0.410 ± 0.082	0.425 ± 0.082	2.250 ± 0.978	2.349 ± 1.014	3.164 ± 0.558	3.242 ± 0.566
	WM	0.327 ± 0.060	0.347 ± 0.061	0.340 ± 0.062	0.345 ± 0.064	1.633 ± 0.759	1.692 ± 0.788	2.498 ± 0.466	2.519 ± 0.466
Dm‐VSASL (1.65/0.35 s)	GM	0.153 ± 0.035	0.431 ± 0.060	0.185 ± 0.040	0.427 ± 0.063	1.191 ± 0.557	2.507 ± 1.116	1.861 ± 0.446	3.468 ± 0.716
	WM	0.259 ± 0.049	0.354 ± 0.046	0.299 ± 0.051	0.352 ± 0.050	1.421 ± 0.689	1.797 ± 0.844	2.343 ± 0.490	2.699 ± 0.515
Dm‐VSASL (1.7/0.3 s)	GM	0.365 ± 0.074	0.411 ± 0.070	0.379 ± 0.072	0.403 ± 0.067	2.195 ± 1.139	2.439 ± 1.162	3.075 ± 0.872	3.335 ± 0.868
	WM	0.273 ± 0.056	0.343 ± 0.052	0.299 ± 0.060	0.339 ± 0.054	1.482 ± 0.830	1.727 ± 0.820	2.282 ± 0.638	2.553 ± 0.571

**FIGURE 5:**
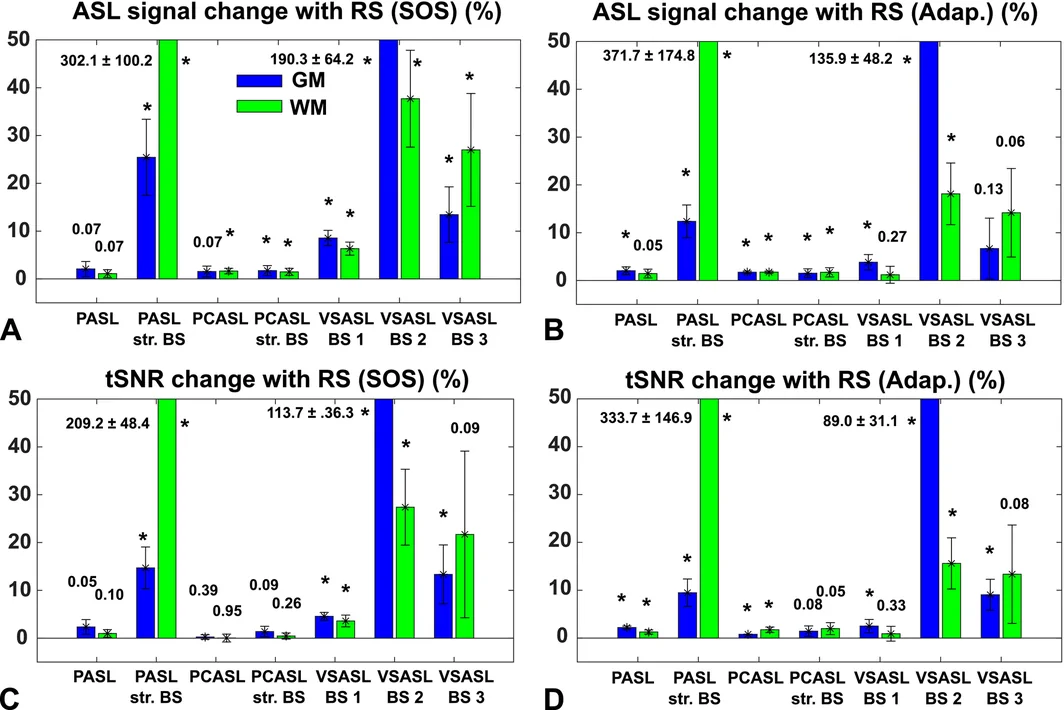
Averaged ASL signal and tSNR percentage changes in gray matter (GM) and white matter (WM) ROIs across subjects when combined with RS‐Recon, under different coil combination and reconstruction environment settings: In‐house SOS coil combination (A and C), and on‐scanner adaptive coil combination (B and D). *p* values from one‐sided *t*‐tests are shown at the top or indicated with asterisks if reached significance (< 0.05); the mean and standard deviation values are shown for the error bars exceeding the plot boundary. PASL, Pulse ASL; PCASL, Pseudo‐continuous ASL; str. BS, strong BS; VSASL, velocity‐selective ASL; BS 1–3: Dm‐VSASL with TI_1_/TI_2_ = 1.6/0.4 s, 1.65/0.35 s, and 1.7/0.3 s.

Examples of ComplexSub and ComplexSub + RS results are shown in Figure 6, in comparison of Mag and Mag + RS. In ComplexSub, because the subtraction was done early in the *k*‐space, despite that temporal pre‐averaging was done on the complex data, subsequent SOS coil combination led to rectification of the signal and noise, resulting in loss of the signal sign and the signal bias, as indicated by the elevated noise background in the signal profiles outside of the brain (Figure 6B). In contrast, when RS‐recon was included, the signs of ASL signal and noise were preserved after removing the superimposed reference signals, resolving the above issues even with SOS coil combination.

**FIGURE 6:**
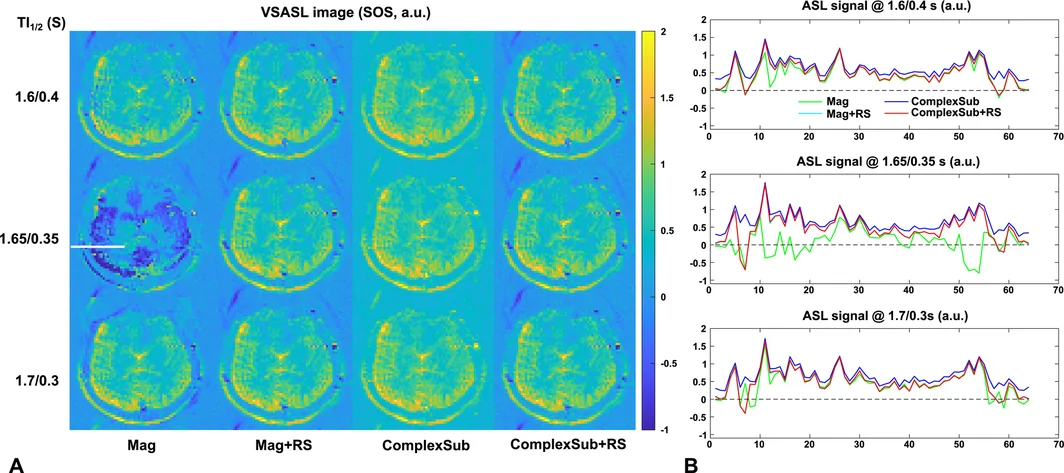
Reconstruction examples comparing SOS based Mag and ComplexSub reconstruction, with and without RS‐recon: (A) Examples of reconstructed dm‐VSASL images under different levels of BS; (B) ASL signal profiles across the image slice along the white line indicated in (A) ASL signal artifacts due to sign‐flipping were corrected with RS or ComplexSub‐based reconstruction. ComplexSub elevated the background signal/noise and lost the signal sign due to SOS coil combination; these were corrected when RS‐recon was included.

Examples of a closer comparison between different ComplexSub‐based methods are shown in Figures 7 and S1. Examples of ComplexSub‐based and other RS‐based reconstruction at individual time points can be found in Figures S2 and S3. Comparing the SOS and adaptive coil combination methods with ComplexSub, SOS resulted in more profoundly elevated noise outside of the brain due to additional signal rectification effects during coil combination. Such effects were more profound if temporal averaging was performed after coil combination and image output (post‐averaging, Figures S2 and S3). Similarly, when magnitude output was used with adaptive coil combination, negative signals/noise were rectified. With the complex signal projection, the sign information was preserved, but regional phase and ASL signal projection errors occurred in low‐SNR regions with self‐estimated coil sensitivity maps for coil combination. In really low‐SNR cases, the adaptive coil combination using self‐estimated sensitivity maps could completely fail, as shown in the examples in Figure S3. Comparing the ComplexSub + adaptive + projection pipelines, when the coil sensitivity was estimated from a high‐SNR reference (*M*_0_), the phase and ASL signal projection errors were corrected. When RS‐recon was included, the signal projection successfully recovered ASL signals with correct signs regardless of the source of the coil sensitivity estimation, demonstrating that the RS‐recon provided good coil sensitivity estimation with improved phase estimation accuracy. This also enables ASL images to be reconstructed at individual time points with desired temporal noise characteristics, such as zero bias and Gaussian distribution instead of Rician. A simple pipeline with RS + SOS also resulted in accurate detection of signed ASL signals with temporal pre‐averaging; however, with post‐averaging, the noise outside of the brain was amplified, even though the signal and noise in the brain seemed unaffected.

**FIGURE 7:**
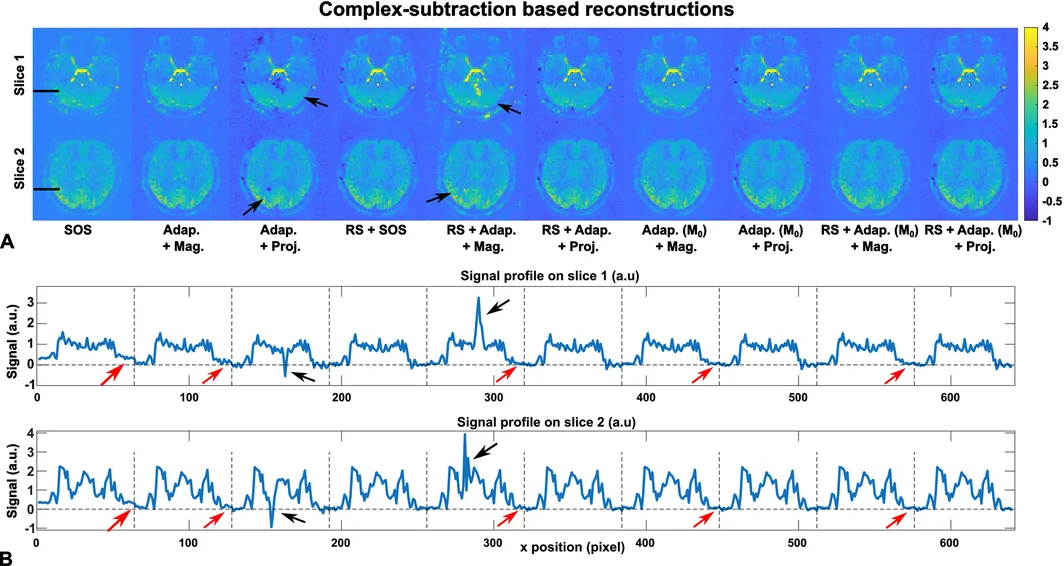
Reconstruction examples of dm‐VSASL using different complex‐subtraction based pipelines: (A) Reconstructed ASL images; (B) ASL signal profiles along the black lines shown in (A) the elevated or rectified background signal/noise, and the signal detection errors are indicated with red and black arrows, respectively.

Given the small number of subjects, no significant difference was detected by ANOVA (*p* > 0.994) among the normalized ASL signal and tSNR in GM and WM using the four representative RS‐based reconstruction pipelines, that is, Mag + RS (SOS), Mag + RS (adaptive combination with magnitude output), ComplexSub + RS (adaptive combination with projection), and Complex + RS (adaptive combination with projection). The averaged percentage differences between any of the comparison pairs were negligible, that is, < 1% for normalized ASL signal and < 0.5% for tSNR, suggesting very similar performance between these RS‐based reconstruction pipelines.

RS‐recon is robust against misalignment between the reference and ASL acquisitions. As shown in Figure 8, in comparison to well‐aligned Mag, Mag + RS yielded improved ASL signal detection and tSNR in GM and WM with at least up to ∼4 mm 3D shift between the reference and PCASL images with a 32‐channel coil. With VSASL, such improvement was observed with up to ∼15 mm misalignment. Such robustness against the misalignment was greater with the 16‐channel coil, likely due to smoother coil sensitivity profiles across the imaging volume compared to the 32‐channel coil. The ASL signal and tSNR in WM were more insensitive to the misalignment than GM; this was expected because the WM ROIs are more clustered while the GM ROIs are more dispersed, making the latter more sensitive to misalignment.

**FIGURE 8:**
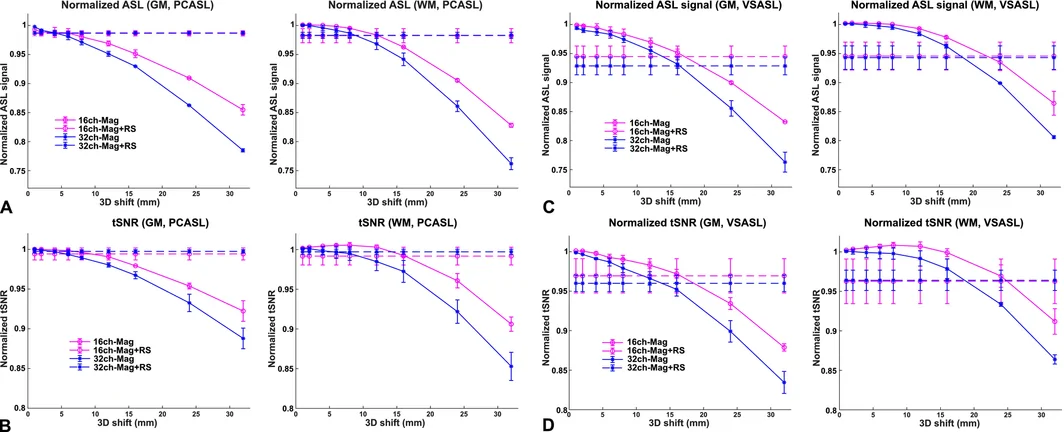
Normalized ASL signal and tSNR in GM and WM with different 3D shifts of the reference (*M*_0_) images, using PCASL (A and B) and VSASL (C and D), and acquired with 32‐ and 16‐channel receive coils. ASL signals and tSNR were normalized to the Mag + RS results without shift. The results without RS‐Recon were plotted at all shifts to serve as reference lines.

Examples of combining RS‐recon with the GRAPPA reconstruction pipeline are shown in Figure 9, demonstrating that RS‐recon can be readily integrated into the existing parallel imaging reconstruction pipelines, such as GRAPPA in this proof‐of‐concept experiment, to correct subtraction errors and reconstruction artifacts using different labeling methods.

**FIGURE 9:**
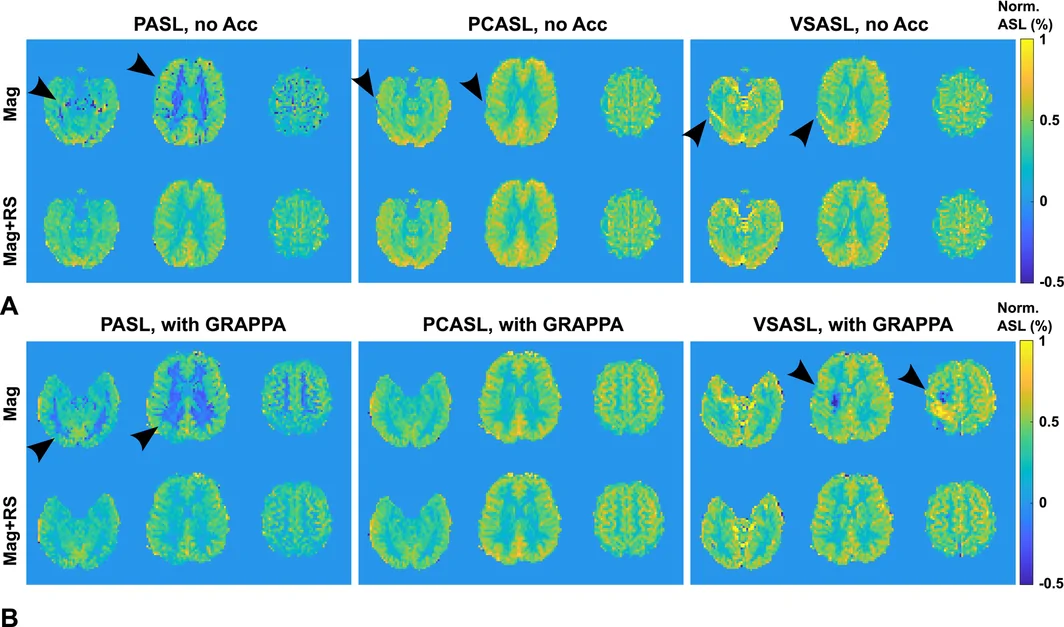
On‐scanner reconstruction results without and with parallel imaging acceleration. (A) Reconstruction results without parallel imaging, showing ASL signal recovery and artifact removal across different ASL labeling methods. The PCASL examples showed correction to a subtle signal loss likely due to phase detection errors where no sign‐flipping was expected. (B) Reconstruction results with GRAPPA parallel imaging acceleration (2× in‐plane) from another subject, showing that RS‐recon can be readily integrated with the parallel imaging reconstruction pipeline. The artifacts due to sign‐flipping and/or phase detection error are indicated with black arrowheads.

## 5 Discussion

In this study, we provided a general and formalized description of the ASL image reconstruction pipeline. With the key components identified as combinable modules, this allows a more precise definition of any specific reconstruction pipeline for ASL. Thanks to the high‐SNR reference data (*m*_0_) acquired routinely in ASL, the new RS‐recon strategy offers the following features and advantages: (1) it completely solves the signal flipping problem in ASL even with magnitude output, which is the most widely used practice; (2) this enables ideal BS to be implemented in ASL for optimal SNR; (3) it preserves the sign of the ASL signals, for example negative values, which may be helpful in understanding the labeling physics or identifying specific information in pathologies; (4) it allows more accurate and robust phase detection for correct signal combination across coil channels; (5) signed tissue signals are available at individual time point (except for ComplexSub + RS), which can be used for motion correction if needed; (6) it prevents signal/noise rectification, and keeps the noise zero‐mean and normally distributed; (7) it provides high‐SNR coil sensitivity maps without a dedicated coil sensitivity scan; (8) it is easy to implement, and readily integrates with existing ASL image reconstruction pipelines, including those using parallel imaging acceleration.

RS‐recon allows ideal BS to be applied in ASL for optimal SNR performance, without worrying about signal cancelation due to sign‐flipping. This significantly relaxes the constraints in BS timing design with more flexibility. For example, the tissue signals are no longer required to be of the same positive sign, which makes suppressing all relevant background tissues more achievable, whereas trying to prioritizing a few major tissue types to suppress may result in suboptimal BS overall. This may also allow fewer BS pulses to be used for optimized BS which better preserves ASL signal and reduces SAR.

In ComplexSub‐based reconstructions, the MR signals obtained at individual time point after subtraction are relatively weak as only ASL signals are present. Conventionally temporal pre‐averaging on the complex data is performed to improve the SNR before coil combination. As demonstrated with the examples in Figure 7, the background signals were slightly increased with SOS or adaptive coil combination with magnitude output, as most of the noise is canceled during the complex‐valued temporal averaging. However, in applications where ASL images at individual time points are of interest, for example, in ASL fMRI experiments, the rectified noise from SOS coil combination or magnitude output can accumulate over time, leading to stronger artifacts and compromised SNR with post‐averaging (Figures S2 and S3). This can be solved with RS‐recon with complex (adaptive) coil combination and signal projection, for example, ComplexSub + RS + SOS with pre‐averaging (Figure 7) and post‐averaging (Figure S2). For ComplexSub + RS + SOS post‐averaging resulted in magnified noise outside of the imaging object, where no high‐SNR reference signal was available to preserve the signal sign/phase; while the ASL signal detection in the brain still benefited from the RS processing with high‐SNR reference signals. Among the ComplexSub‐based reconstruction, RS combined with adaptive coil combination and signal projection is recommended for its robustness and accuracy; if pre‐averaging is used, that is, ASL images at individual time points are not needed, RS + SOS may also be considered for its simplicity and robustness.

Comparing the pipelines of different types, ComplexSub was originally designed to overcome the sign‐flipping issue with magnitude output, but can result in biased and rectified ASL signal estimation. In this study we have shown that this issue can be solved by RS‐recon when combining with adaptive coil combination and complex signal projection. Comparing the RS‐integrated methods that require adaptive coil combination and complex signal projection, that is, ComplexSub + RS and Complex + RS, Complex + RS may be a preferred method for its more straightforward implementation and the capability to yield individual label/control images for motion correction, same as with Mag‐based pipelines. On the other hand, even with the simplest implementation using SOS coil combination the Mag + RS strategy offered robust performance that was almost identical to ComplexSub + RS and Complex + RS with adaptive coil combination and complex signal projection. Because RS‐recon avoid signal sign‐flipping, even with magnitude output the noise after label/control subtraction remains zero‐mean and normally distributed (see examples in Figure S4). In addition, the magnitude output form is the most widely adopted and would likely integrate seamlessly with the current imaging processing workflows in various research and clinical settings. Therefore, Mag + RS is the most recommended reconstruction strategy for ASL overall.

For a successful implementation of the RS‐recon for the GRASE‐EPI readout used here, we found that it was also necessary to apply the RS‐recon principle on the EPI‐reference line acquisition for Nyquist ghosting correction, that is, superimposing the *k*‐space data of the reference lines from the *M*_0_ acquisition to those in each individual ASL acquisition. This is because when the MR signal is low, the phase information estimated from the low‐SNR reference lines for Nyquist ghosting correction may be inaccurate, and not suitable for correcting the superimposed ASL *k*‐space data.

Though the focus of this study was not on comparing different BS conditions, the results, for example, using PCASL, showed that the improved tSNR gain outweighed the ASL signal reduction due to the use of additional BS pulses, supporting the recommendation of applying BS whenever possible [3].

The effectiveness of RS‐recon has been demonstrated in ASL in this study; the principle should be readily applicable to and benefit other MRI applications where the target MR signal has low SNR but a high‐SNR reference scan is available, such as diffusion imaging.

Finally, there are limitations in the current study that can be further investigated: (1) we were able to freely adjust the BS to cover more representative and relevant BS conditions for dm‐VSASL, but were not able to do so for PASL and PCASL due to limited BS options; (2) the number of subjects was small, though it was sufficient to demonstrate the main properties of the technique, including more subjects would help improve the statistical power regarding its performance using different reconstruction pipelines, and potentially making useful recommendations with greater confidence; (3) for its integration with parallel imaging, we only tested it with GRAPPA which is a *k*‐space based technique as a proof of concept; though it is expected that RS‐recon should work with other types of parallel imaging techniques, including those operating in image space, it needs to be tested; (4) it was only demonstrated with EPI readout in the current study, though we expect that the RS‐Recon principle should be generalizable to other readout trajectories, such as spiral, radial, stack‐of‐stars, etc., this needs to be validated; (5) for simplicity and demonstration purposes only 3D diagonal shifts were tested, its robustness against rotational misalignment should be evaluated in future work.

## 6 Conclusion

We have developed a new reconstruction strategy, reference‐superimposed reconstruction, for ASL imaging. It uses the high‐SNR *M*_0_ reference data for robust and error‐free ASL image reconstruction. Through theoretical analysis and in vivo experiments, we have shown that this new strategy solves the sign‐flipping problem even with magnitude‐based reconstruction, enabling ideal background suppression to be applied in ASL, which should offer optimal SNR and temporal stability. RS‐recon works with all labeling methods, is easy to implement, integrates well with all existing ASL reconstruction pipelines including parallel imaging, and is robust against misalignment between the reference and ASL scans. Overall, Mag + RS is the most recommended reconstruction strategy for ASL for its simplicity, robustness, and compatibility overall.

## Funding

This work was supported by the National Institutes of Health, R01CA284172 and R01EB033210.

## Conflicts of Interest

The author declares no conflicts of interest.

## Supporting information


**Table S1:** Normalized ASL signal (%) in gray matter (GM) and white matter (WM) using different reconstruction pipelines and reconstruction environments, averaged across subjects (mean ± standard deviation, std).
**Table S2:** Temporal SNR (tSNR) in gray matter (GM) and white matter (WM) using different reconstruction pipelines and reconstruction environments, averaged across subjects (mean ± standard deviation, std).
**Figure S1:** Reconstruction examples of PCASL using different complex‐subtraction based pipelines, similar to those shown in Figure 7: (A) reconstructed ASL images; (B) ASL signal profiles along the black lines shown in (A). The elevated or rectified background signal/noise, and the signal detection errors are indicated with red and black arrows, respectively.
**Figure S2:** Reconstructed dm‐VSASL time series using complex‐subtraction based pipelines (text in blue), with the results using Mag + RS + SOS (text in black) and Complex + RS + adap.+proj. (text in red) for reference. “Adap. (*M*_0_)” indicates that the adaptive coil combination was performed using high‐SNR sensitivity maps estimated from the *M*_0_ images. Elevated background noise outside of the brain (black arrow) can be observed with Complexsub + RS + SOS after post‐averaging; while the ASL signals in the brain were not affected. The ASL signal errors from the magnitude output and post‐averaging are indicated with red arrows, and the artifacts due to low‐SNR adaptive coil combination with white arrow.
**Figure S3:** Similar to that shown in Figure S2; except that these were from a PCASL scan with low‐SNR using complex‐subtraction based pipelines (text in blue), with Mag + RS + SOS (text in black) and Complex +RS + adaptive + projection (text in red) for reference. “Adap. (*M*_0_)” indicates that the adaptive coil combination was performed using high‐SNR sensitivity maps estimated from the *M*_0_ images. Elevated background noise outside of the brain (black arrow) can be observed with Complexsub + RS + SOS after post‐averaging; while the ASL signals in the brain were not affected. The ASL signal errors from the magnitude output and post‐averaging are indicated with red arrows. In this example, adaptive coil combination failed at both individual time point and post‐averaging stages due to low SNR (white arrows).
**Figure S4:** Histograms of voxels in a noisy ROI outside of the brain in averaged ASL images from a PCASL scan (A) and a dm‐VSASL scan (B), using Mag and Mag + RS reconstruction pipelines with adaptive coil combination and magnitude output. The noise distributions were similar without and with RS‐recon, both were normal (verified by Jarque‐Bera normality test).

## Data Availability

The Python codes to superimpose *M*_0_ raw *k*‐space data onto ASL raw *k*‐space data are available through the Open Science Framework DOI: https://doi.org/10.17605/OSF.IO/AE9NU.
