# Supplementary material for: Reference‐Superimposed Reconstruction (RS‐Recon) for Arterial Spin Labeling
Source: Magn Reson Med. 2026 Jul 26;96(6):2685–97. doi: 10.1002/mrm.70540 (PMC13619874; doi:10.1002/mrm.70540)
Supplement: Supplementary file 1 [file MRM-96-2685-s001.pdf]

Supporting Information

Mathematical derivation for Reference-superimposed reconstruction (RS-recon)

For a given coil channel, let the tissue signal S_t and the blood signal S_b under the ASL acquisition, and the reference signal S_{m_0} acquired under the calibration/reference scan be real: $S_t, S_b, S_{m_0} \in R$, i.e. they are in phase.

The measured ASL raw signal is

$S_{ASL} = S_t + S_b + e$,

where e is the error term with a fixed amplitude ϵ and an independent uniform phase $\theta \sim U[0, 2\pi)$, i.e., $e = \epsilon e^{j\theta}$.

We assume the noise in the reference scan follows the same distribution as in the ASL scan. Then, the reference signal is:

$S_{Ref} = S_{m_0} + e$.

And the signal using RS-recon is:

$S_{RS} = S_{ASL} + \alpha S_{Ref}$, where α is a real scaling factor defined by the user; for simplicity, we assume α is positive in the following discussion.

For preventing signal sign-flipping:

Given that the tissue and the blood signals can both be negative, or have opposite signs under the label and control conditions, it needs to satisfy

$\alpha |S_{m_0}| > |S_t| + |S_b|$,

to avoid signal sign-flipping**.**

For example, given a background suppression (BS) level of 10%, i.e. $|S_t| \approx 0.1 |S_{m_0}|$, and $|S_b| \approx 0.01 |S_{m_0}|$ in the brain, for any $\alpha > 0.11$ signal sign-flipping in magnitude-based reconstruction can be prevented. When a stronger BS is used, this can be achieved with lower α values.

For improved phase detection:

For simplicity, we assume that S_t, S_b, S_{m_0} are all positive with a true phase $\phi_{true} = 0$, and that the error magnitude is small relative to the signal magnitude, then the original phase estimation error can be approximated as

$$\Delta\phi_{orig} \approx \frac{Im(S_{ASL})}{Re(S_{ASL})} = \frac{Im(e)}{S_t + S_b + Re(e)} \approx \frac{Im(e)}{S_t + S_b},$$

Where it has a mean of 0, i.e. $E[\Delta\phi_{orig}] = 0$, and a variance

$Var(\Delta\phi_{orig}) = E[\Delta\phi_{orig}{}^2] = \frac{\epsilon^2}{2(S_t + S_b)^2}$, due to independent uniform phases.

Thus, the mean squared error (MSE) of the original phase estimation from the ASL signal is:

$$MSE_{orig} = E[\Delta\phi_{orig}{}^2] = \frac{\epsilon^2}{2(S_t + S_b)^2}.$$

With RS-recon,

$$S_{RS} = S_{ASL} + \alpha S_{Ref} = S_t + S_b + e + \alpha S_{m_0} + \alpha e.$$

Now we consider two scenarios:

1. Multiple independent reference scans are used.

Under this scenario, the MSE of the phase estimation from RS-recon can be derived similarly as:

$$MSE_{RS} = E[\Delta\phi_{RS}{}^2] \approx \frac{(1+\alpha^2)\epsilon^2}{2(S_t + S_b + \alpha S_{m_0})^2}.$$

The ratios of the MSE with RS-recon to that without is:

$$\frac{MSE_{RS}}{MSE_{orig}} = \frac{(1+\alpha^2)(S_t + S_b)^2}{(S_t + S_b + \alpha S_{m_0})^2}.$$

Let $S_{m_0} = \beta(S_t + S_b)$, where β is the BS factor, roughly equals 1/(BS level), e.g., with a BS level of 10%, $\beta \approx 10$. It is a positive ratio greater than 1, as ASL scans have lower signals than that at equilibrium.

Then, the phase estimation accuracy is improved with RS-recon:

$$\frac{MSE_{RS}}{MSE_{orig}} = \frac{(1+\alpha^2)(S_t + S_b)^2}{(S_t + S_b + \alpha S_{m_0})^2} = \frac{1+\alpha^2}{(1+\alpha\beta)^2} < 1, \text{ for any positive } \alpha.$$

2. A single reference scan is superimposed.

This is the case how RS-recon is proposed to be implemented. Under this scenario, a fixed signal from the reference scan, i.e., $S_{Ref} = S_{m_0} + e_0 = S_{m_0} + \epsilon e^{j\theta_0}$ where θ_0 is the measured phase from the reference scan, is superimposed to ASL data acquired at multiple time points. This contributes a non-zero bias and a zero variance to the estimation:

Bias: $E[\Delta\phi_{RS}] = \frac{\alpha Im(e_0)}{S_t + S_b + \alpha S_{m_0} + Re(e) + \alpha Re(e_0)} \approx \frac{\alpha Im(e_0)}{S_t + S_b + \alpha S_{m_0}}$, and $|E[\Delta\phi_{RS}]| \leq \frac{\alpha\epsilon}{S_t + S_b + \alpha S_{m_0}}$, as $|Im(e_0)| \leq \epsilon$;

Variance: $Var(\Delta\phi_{RS}) = E\left[\Delta\phi_{RS}{}^2\right] \approx \frac{\epsilon^2}{2(S_t + S_b + \alpha S_{m_0})^2}$.

Thus, the MSE of the phase estimation using RS-recon is:

$MSE_{RS} = Var + Bias^2 = \frac{\epsilon^2 + 2\alpha^2 Im(e_0)^2}{2(S_t + S_b + \alpha S_{m_0})^2}$.

Since $|Im(e_0)| \leq \epsilon$, we have a tight bound:

$MSE_{RS} \leq \frac{(1+2\alpha^2)\epsilon^2}{2(S_t + S_b + \alpha S_{m_0})^2}$,

and the MSE ratio is

$\frac{MSE_{RS}}{MSE_{orig}} \leq \frac{(1+2\alpha^2)(S_t + S_b)^2}{(S_t + S_b + \alpha S_{m_0})^2}$.

With $S_{m_0} = \beta(S_t + S_b)$, we have:

$\frac{MSE_{RS}}{MSE_{orig}} \leq \frac{1+2\alpha^2}{(1+\alpha\beta)^2}$.

For a phase estimation with improved accuracy, i.e. $\frac{MSE_{RS}}{MSE_{orig}} < 1$, we have $\frac{1+2\alpha^2}{(1+\alpha\beta)^2} < 1$, or $(2-\beta^2)\alpha < 2\beta$. For $\beta \geq \sqrt{2}$, the inequality holds for all positive α; for 1< $\beta \leq \sqrt{2}$, it holds when $\alpha < \frac{2\beta}{(2-\beta^2)}$. The minimum of $\frac{MSE_{RS}}{MSE_{orig}}$, i.e. the highest accuracy, is reached when $\alpha = \beta/2$ by solving $\frac{d\frac{MSE_{RS}}{MSE_{orig}}}{d\alpha} = 0$.

For a higher β, e.g., ASL scans with a stronger BS, the ratio plateaued earlier, so a much lower α is needed to approximate the minimum, as shown in **Figure 2** in the paper. And keeping α low helps reduce the estimation bias.

Supporting Information Table S1. Normalized ASL signal (%) in gray matter (GM) and white matter (WM) using different reconstruction pipelines and reconstruction environments, averaged across subjects (mean ± standard deviation, std).

		In-house recon. in Matlab						On-scanner recon.			
Norm. ASL (%, mean ± std)		Mag (SOS)	Mag+RS (SOS)	ComplexSub (Adap+Proj)	ComplexSub +RS (Adap+Proj)	Complex (Adap+Proj)	Complex +RS (Adap+Proj)	Mag (Adap)	Mag+RS (Adap)	Complex (Adap+Proj)	Complex+RS (Adap+Proj)
PASL (BS)	GM	0.346 ± 0.068	0.353 ± 0.066	0.193 ± 0.177	0.350 ± 0.066	0.340 ± 0.068	0.351 ± 0.065	0.328 ± 0.065	0.334 ± 0.064	--	--
	WM	0.274 ± 0.048	0.277 ± 0.046	0.156 ± 0.134	0.276 ± 0.047	0.270 ± 0.048	0.276 ± 0.046	0.255 ± 0.045	0.257 ± 0.045	--	--
PASL (BS-strong)	GM	0.273 ± 0.067	0.339 ± 0.064	0.182 ± 0.156	0.337 ± 0.063	0.321 ± 0.065	0.338 ± 0.063	0.288 ± 0.064	0.319 ± 0.064	--	--
	WM	0.069 ± 0.021	0.265 ± 0.051	0.149 ± 0.129	0.264 ± 0.051	0.247 ± 0.055	0.265 ± 0.051	0.058 ± 0.024	0.245 ± 0.049	--	--
PCASL (BS)	GM	0.451 ± 0.090	0.458 ± 0.090	0.290 ± 0.206	0.454 ± 0.090	0.447 ± 0.091	0.454 ± 0.089	0.453 ± 0.089	0.460 ± 0.090	--	--
	WM	0.359 ± 0.058	0.365 ± 0.059	0.237 ± 0.154	0.364 ± 0.060	0.358 ± 0.062	0.365 ± 0.060	0.355 ± 0.058	0.360 ± 0.059	--	--
PCASL (BS-strong)	GM	0.403 ± 0.079	0.410 ± 0.076	0.229 ± 0.205	0.408 ± 0.076	0.394 ± 0.079	0.408 ± 0.075	0.415 ± 0.076	0.419 ± 0.076	--	--
	WM	0.327 ± 0.055	0.332 ± 0.054	0.198 ± 0.160	0.331 ± 0.054	0.324 ± 0.054	0.332 ± 0.054	0.330 ± 0.051	0.332 ± 0.051	--	--
Dm-VSASL (1.6/0.4s)	GM	0.393 ± 0.077	0.426 ± 0.078	0.272 ± 0.209	0.425 ± 0.079	0.397 ± 0.079	0.425 ± 0.079	0.410 ± 0.082	0.425 ± 0.082	0.426 ± 0.081	0.425 ± 0.081
	WM	0.327 ± 0.060	0.347 ± 0.061	0.233 ± 0.159	0.346 ± 0.060	0.325 ± 0.066	0.345 ± 0.060	0.340 ± 0.062	0.345 ± 0.064	0.350 ± 0.063	0.346 ± 0.064
Dm-VSASL (1.65/0.35s)	GM	0.153 ± 0.035	0.431 ± 0.060	0.248 ± 0.199	0.431 ± 0.060	0.353 ± 0.065	0.431 ± 0.060	0.185 ± 0.040	0.427 ± 0.063	0.419 ± 0.060	0.429 ± 0.063
	WM	0.259 ± 0.049	0.354 ± 0.046	0.216 ± 0.145	0.353 ± 0.046	0.287 ± 0.059	0.353 ± 0.046	0.299 ± 0.051	0.352 ± 0.050	0.355 ± 0.054	0.353 ± 0.050
Dm-VSASL (1.7/0.3s)	GM	0.365 ± 0.074	0.411 ± 0.070	0.259 ± 0.176	0.412 ± 0.071	0.363 ± 0.072	0.411 ± 0.071	0.379 ± 0.072	0.403 ± 0.067	0.406 ± 0.069	0.408 ± 0.070
	WM	0.273 ± 0.056	0.343 ± 0.052	0.220 ± 0.138	0.342 ± 0.051	0.316 ± 0.050	0.342 ± 0.052	0.299 ± 0.060	0.339 ± 0.054	0.335 ± 0.055	0.340 ± 0.054

Supporting Information Table S2. Temporal SNR (tSNR) in gray matter (GM) and white matter (WM) using different reconstruction pipelines and reconstruction environments, averaged across subjects (mean ± standard deviation, std).

		In-house recon.						On-scanner recon.			
Norm. ASL (%, mean ± std)		Mag (SOS)	Mag+RS (SOS)	ComplexSub (Adap+Proj)	ComplexSub +RS (Adap+Proj)	Complex (Adap+Proj)	Complex +RS (Adap+Proj)	Mag (Adap)	Mag+RS (Adap)	Complex (Adap+Proj)	Complex+RS (Adap+Proj)
PASL (BS)	GM	1.875 ± 1.051	1.912 ± 1.059	1.210 ± 1.356	1.910 ± 1.062	1.854 ± 1.057	1.911 ± 1.059	2.758 ± 0.971	2.810 ± 0.985	--	--
	WM	1.416 ± 0.786	1.426 ± 0.784	0.842 ± 0.913	1.426 ± 0.785	1.394 ± 0.783	1.427 ± 0.784	2.181 ± 0.676	2.203 ± 0.678	--	--
PASL (BS-strong)	GM	1.530 ± 0.827	1.729 ± 0.882	1.023 ± 1.110	1.723 ± 0.882	1.676 ± 0.874	1.727 ± 0.882	2.363 ± 0.753	2.558 ± 0.773	--	--
	WM	0.454 ± 0.274	1.335 ± 0.713	0.762 ± 0.816	1.334 ± 0.712	1.277 ± 0.729	1.337 ± 0.712	0.544 ± 0.249	2.112 ± 0.549	--	--
PCASL (BS)	GM	1.737 ± 0.721	1.741 ± 0.724	1.159 ± 1.097	1.733 ± 0.723	1.727 ± 0.726	1.736 ± 0.722	2.352 ± 0.606	2.368 ± 0.612	--	--
	WM	1.577 ± 0.660	1.579 ± 0.665	1.042 ± 0.991	1.573 ± 0.664	1.562 ± 0.668	1.576 ± 0.663	2.273 ± 0.462	2.306 ± 0.467	--	--
PCASL (BS-strong)	GM	2.369 ± 1.158	2.392 ± 1.149	1.461 ± 1.604	2.388 ± 1.153	2.312 ± 1.160	2.390 ± 1.151	3.647 ± 0.837	3.680 ± 0.830	--	--
	WM	1.886 ± 0.956	1.891 ± 0.954	1.186 ± 1.274	1.891 ± 0.953	1.849 ± 0.949	1.892 ± 0.952	2.999 ± 0.633	3.034 ± 0.620	--	--
Dm-VSASL (1.6/0.4s)	GM	2.250 ± 0.978	2.349 ± 1.014	1.546 ± 1.606	2.350 ± 1.019	2.239 ± 1.005	2.351 ± 1.016	3.164 ± 0.558	3.242 ± 0.566	3.257 ± 0.566	3.249 ± 0.567
	WM	1.633 ± 0.759	1.692 ± 0.788	1.137 ± 1.134	1.690 ± 0.789	1.608 ± 0.777	1.689 ± 0.786	2.498 ± 0.466	2.519 ± 0.466	2.562 ± 0.471	2.530 ± 0.466
Dm-VSASL (1.65/0.35s)	GM	1.191 ± 0.557	2.507 ± 1.116	1.526 ± 1.618	2.509 ± 1.121	2.136 ± 1.080	2.510 ± 1.119	1.861 ± 0.446	3.468 ± 0.716	3.475 ± 0.712	3.477 ± 0.718
	WM	1.421 ± 0.689	1.797 ± 0.844	1.061 ± 1.050	1.797 ± 0.845	1.488 ± 0.781	1.796 ± 0.844	2.343 ± 0.490	2.699 ± 0.515	2.746 ± 0.533	2.712 ± 0.516
Dm-VSASL (1.7/0.3s)	GM	2.195 ± 1.139	2.439 ± 1.162	1.578 ± 1.588	2.441 ± 1.166	2.154 ± 1.141	2.443 ± 1.165	3.075 ± 0.872	3.335 ± 0.868	3.341 ± 0.875	3.346 ± 0.871
	WM	1.482 ± 0.830	1.727 ± 0.820	1.094 ± 1.071	1.728 ± 0.821	1.591 ± 0.838	1.728 ± 0.820	2.282 ± 0.638	2.553 ± 0.571	2.552 ± 0.578	2.567 ± 0.572

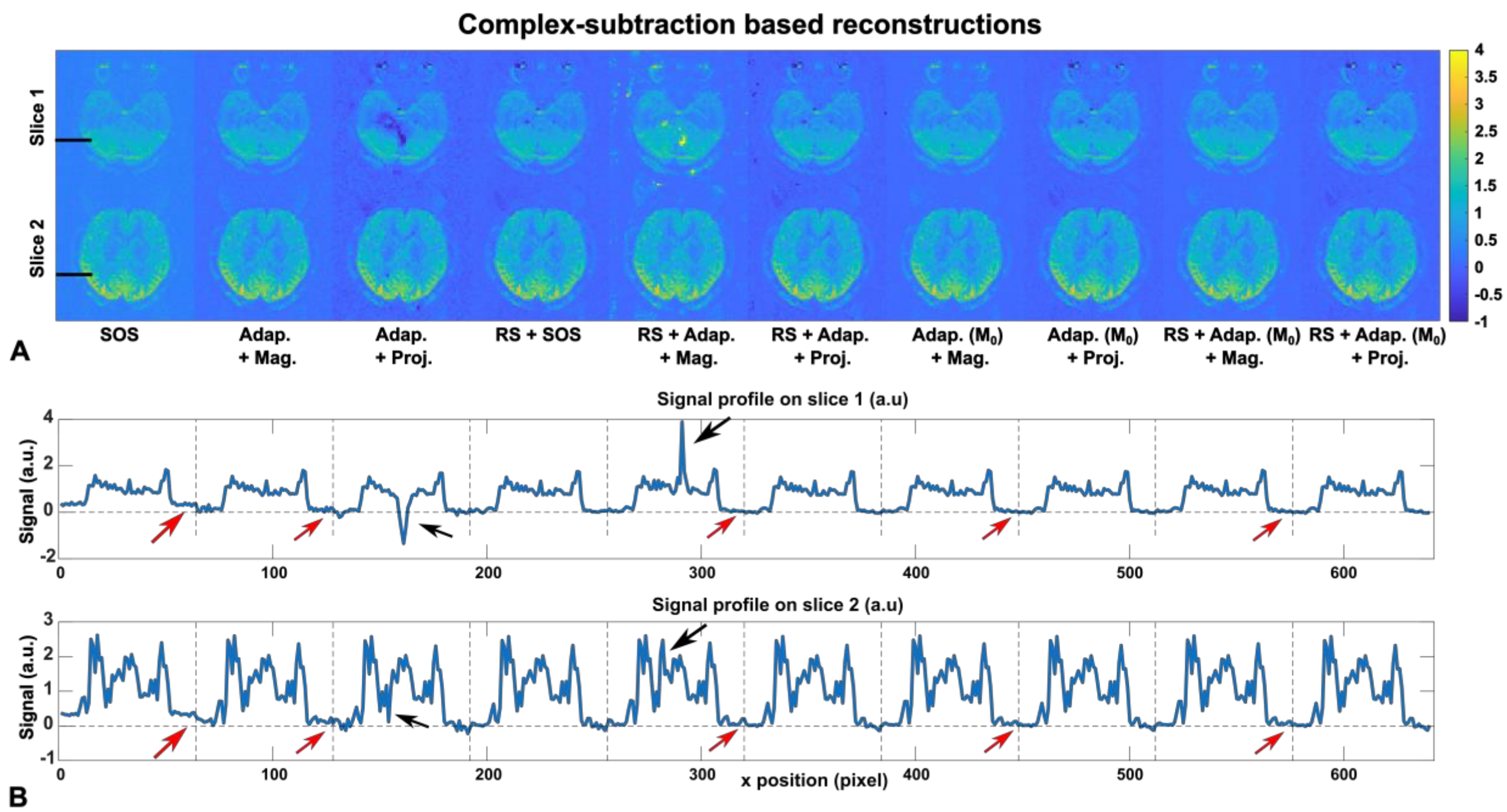

Supporting Information Figure S1. Reconstruction examples of PCASL using different complex-subtraction based pipelines, similar to those shown in **Figure 7**: A. Reconstructed ASL images; B. ASL signal profiles along the black lines shown in A. The elevated or rectified background signal/noise, and the signal detection errors are indicated with red and black arrows, respectively.

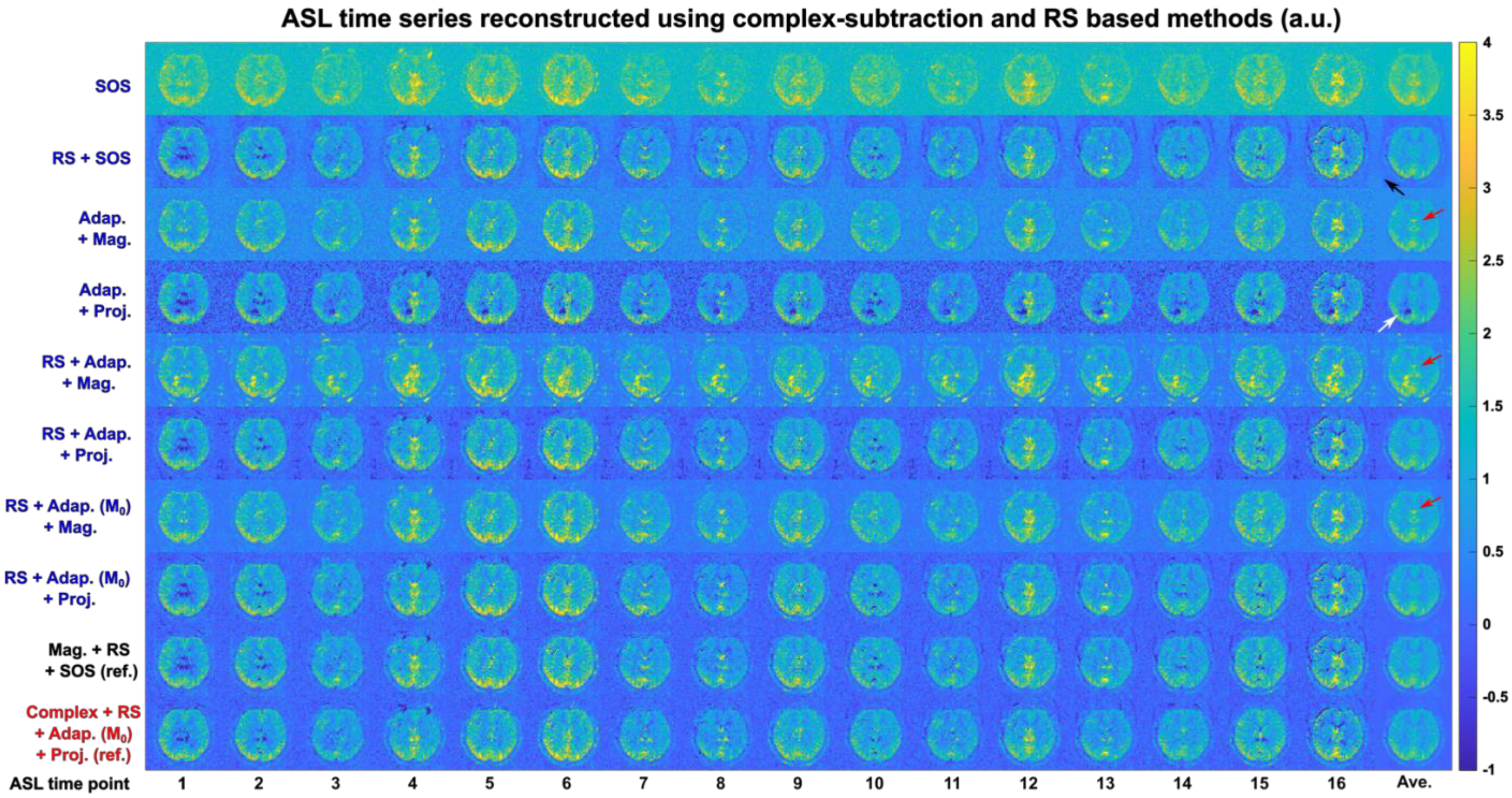

Supporting Information Figure S2. Reconstructed dm-VSASL time series using complex-subtraction based pipelines (text in blue), with the results using Mag+RS+SOS (text in black) and Complex+RS+adap.+proj. (text in red) for reference. “Adap. (M_0)” indicates that the adaptive coil combination was performed using high-SNR sensitivity maps estimated from the M_0 images. Elevated background noise outside of the brain (black arrow) can be observed with Complexsub+RS+SOS after post-averaging; while the ASL signals in the brain were not affected. The ASL signal errors from the magnitude output and post-averaging are indicated with red arrows, and the artifacts due to low-SNR adaptive coil combination with white arrow.

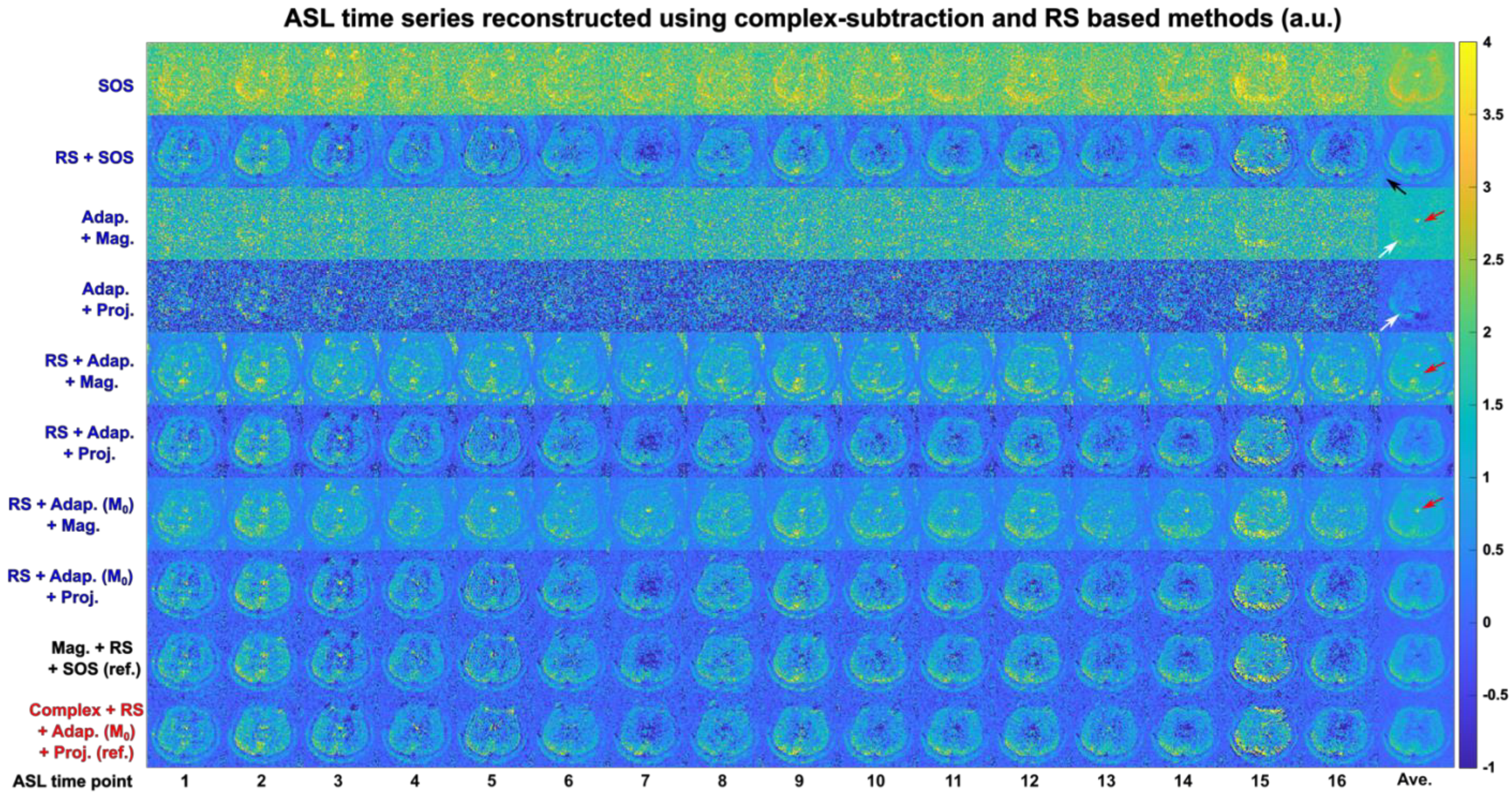

Supporting Information Figure S3. Similar to that shown in **Figure S2**; except that these were from a PCASL scan with low-SNR using complex-subtraction based pipelines (text in blue), with Mag+RS+SOS (text in black) and Complex+RS+adaptive+projection (text in red) for reference. "Adap. (M_0)" indicates that the adaptive coil combination was performed using high-SNR sensitivity maps estimated from the M_0 images. Elevated background noise outside of the brain (black arrow) can be observed with Complexsub+RS+SOS after post-averaging; while the ASL signals in the brain were not affected. The ASL signal errors from the magnitude output and post-averaging are indicated with red arrows. In this example, adaptive coil combination failed at both individual time point and post-averaging stages due to low-SNR (white arrows).

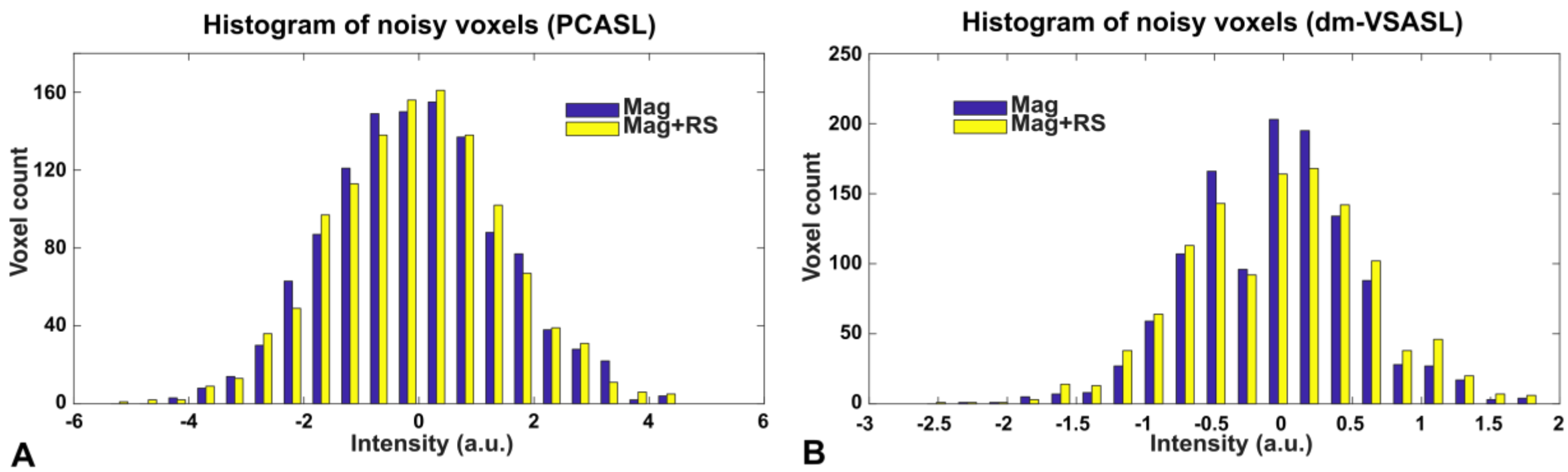

Supporting Information Figure S4. Histograms of voxels in a noisy ROI outside of the brain in averaged ASL images from a PCASL scan (A) and a dm-VSASL scan (B), using Mag and Mag+RS reconstruction pipelines with adaptive coil combination and magnitude output. The noise distributions were similar without and with RS-recon, both were normal (verified by Jarque-Bera normality test).
